# The National Association of Dental Faculties and the National Association of Dental Examiners

**Published:** 1891-09

**Authors:** 


### THE NATIONAL ASSOCIATION OF DENTAL FACULTIES AND THE NATIONAL ASSOCIATION OF DENTAL EXAMINERS.

These two closely-allied bodies met at Saratoga, the first convening August 1, and the latter August 3.

It was not expected that any marked advance would be made by the first-named organization; indeed, it was regarded by the majority of the delegates as unwise to make any move in the direction of an increase of the standard at the present time. The three years' course, which goes into effect this year, was regarded as sufficient until it had been thoroughly tested. Hence the meeting was largely devoted to strengthening lines of present work.

This meeting had been anticipated with some anxiety, as it was feared the harmony heretofore existing between the two bodies would be shattered. The peculiar action of the National Association of Dental Examiners, at Excelsior Springs, Mo., created a feeling of distrust, and the subsequent proceedings of some of the State Boards had evolved a feeling of antagonism in the minds of many connected with college Faculties, that promised nothing but evil results for the future to dental education in this country.

It was hoped, however, that this might be averted by an effort to harmonize differences, and the Association of Faculties very early adopted a resolution appointing a committee of five to confer with a similar committee of the National Association of Examiners. This was accepted by that organization and the joint committee met. The "Faculties," through one of their number, proposed a basis for compromise, as follows:

> *Resolved*, That we recommend to the National Association of Examiners that they take measures to have the laws of the several States so modified that a diploma from a recognized college shall be accepted, provided said college shall furnish the State Board with the standing of the individual holding such diploma. The statement of the college shall comprise the percentage received in each branch. If this fails to satisfy the Board, the examination provided for by the existing laws shall follow, and in response to a request *from* the college the State Board shall furnish the Faculty with a detailed statement of the subjects and questions on which the applicant has failed.

This was adopted by the committee of the Association of Dental Faculties and rejected by that of the National Association of Examiners. They positively refused to adopt anything that might seem to be a recognition of the college diploma. This document, secured at so much cost of time, labor, and money, was to them worthless so far as permitting the holder thereof to the right to practise, and they refused absolutely to be a party to any compromise in this direction.

The temper of the National Association of Examiners, as manifested through their committee, was not of a character to bring about harmony; indeed, it had the reverse effect, and at one period it appeared as though there was nothing for the colleges to do but to carry the matter to the court of last resort. Better counsels prevailed, and a truce was arranged for the present, as follows:

> *Resolved*, That it is recommended to the State Boards that when a graduate, after examination, has been refused a license and his college requests information as to the cause of his failure to pass the examination, the Boards shall furnish the Faculty with a detailed statement of the subjects and questions on which the applicant has failed.
>
> *Resolved*, That we discountenance the publication by the State Boards of the names of colleges whose graduates have failed to pass.

We cannot regard the question as settled by the wording of the resolution. It would be a satisfaction to be able to feel that these two powerful organizations were in perfect unison. No good result can be obtained by a state of war. The interests of all are jeopardized by the present antagonistic position. The colleges, as we understand it, recognize the importance of maintaining laws, but they hold that these should be developed as the needs of both colleges and the public are made apparent. The sure evolutionary process is always a slow one, and State Boards cannot expect to overthrow a general law by an attack along all the lines with a force simply destructive. The colleges have made the profession, not the latter the colleges, and it seems to us that it is in the last degree inopportune, if not an exhibition of ingratitude, that the Boards of Examiners should now endeavor, through force of law, to crush the hand that has made their existence possible. It will, probably, be denied that this is the intention, and we have no doubt but that the motives are good, but the action is certainly in the wrong direction, and can have, if persisted in, but one result, the sapping of the life of the schools.

If, as has been stated, the present laws prevent the accepting of the compromise measure as originally presented by the National Association of Faculties, then it is certainly time that efforts should be made the coming winter to have the laws amended.

It may be stated that no legislation will be satisfactory that arranges for the appointment of State Boards through political influence, or these bodies at all, unless there is some certainty that they represent the highest culture of the profession. How this is to be tested remains for future developments, but until something better than we have at present is proposed, boards of examiners should hesitate in introducing forceful measures.

